# The Prevalence of Anemia in Hospitalized Patients With Diabetic Foot Ulcer (DFU) and the Relationship Between the Severity of Anemia and the Severity of DFU

**DOI:** 10.7759/cureus.41922

**Published:** 2023-07-15

**Authors:** Ritesh Kumar, Surya K Singh, Neeraj K Agrawal, Ujwal Kumar, Subhash Kumar, Supreeth C, Avina Bishnoi

**Affiliations:** 1 Department of Endocrinology and Metabolism, Institute of Medical Sciences, Banaras Hindu University, Varanasi, IND; 2 Department of Urology, Institute of Medical Sciences, Banaras Hindu University, Varanasi, IND

**Keywords:** anemia, hemoglobin, anemia of chronic disease, infection, type 2 dm, diabetic foot ulcer

## Abstract

Background and aims: We aim to determine the prevalence of anemia in hospitalized patients with diabetic foot ulcers (DFUs) and estimate the relationship between the severity of anemia and diabetic foot ulcer.

Materials and methods: We retrospectively collected and evaluated the data of 323 patients hospitalized with diabetic foot ulcer (DFU). We included 299 type 2 diabetic patients with foot ulcers of neuropathic or neuroischemic nature with infection. Anemia was defined based on World Health Organization (WHO) criteria, and the severity of DFU was classified in University of Texas (UT) grades.

Results: Anemia was detected in 94.3% of DFU, and the prevalence of mild, moderate, and severe anemia was 16.7%, 55.7%, and 27.6%, respectively. There was a significant difference in the mean hemoglobin (Hb) levels among the patients with varying grades of severity of DFU (1B: Hb=10.17±2.08 gm/dL, 2B: Hb=9.27±2.04 gm/dL, 3B: Hb=8.03±1.829 gm/dL; p value=<0.0001). The iron study was available in 141 (47.15%) patients and was suggestive of anemia of chronic disorder (mean serum iron=40.22±23.81 mcg/dL, mean total iron-binding capacity (TIBC)=239.34±67.24 mcg/dL, mean ferritin=378.05±141.337 ng/mL). TIBC significantly decreased (1B=262.13±61.05, 2B=233.65±71.26, 3B=222.43±74.18; p=0.04), and ferritin significantly increased (1B=309.9±70.76, 2B=351.73±94.22, 3B=488.58±170.4; p<0.0001) with increasing DFU severity. Hemoglobin was significantly decreased at the time of discharge in comparison to that at admission (9.3±2.1 gm/dL versus 8.8±1.5 gm/dL; p value=0.01). Red blood cell (RBC) counts, mean corpuscular volume (MCV), mean corpuscular hemoglobin concentration (MCHC), lymphocyte counts, albumin, calcium, and high-density lipoprotein (HDL) significantly decreased with the increase of DFU severity. The duration of hospitalization, total leucocyte counts, neutrophil counts, and neutrophil-to-lymphocyte ratio (NLR) increased with the severity of DFU.

Conclusions: The prevalence of anemia was very high in DFU and more than three-fourths of the patients had moderate to severe anemia. The severity of anemia was associated with the severity of DFU. The most common cause of anemia was anemia of chronic disorder secondary to diabetic foot infection. During the period of hospitalization, hemoglobin decreased despite improvement in DFU infection.

## Introduction

The prevalence of diabetes mellitus (DM) has increased at an alarming rate worldwide and contributes to significant morbidity and mortality in affected individuals. One of the serious complications of diabetes is diabetic foot ulcer (DFU), which leads to frequent and prolonged hospitalization. The prevalence of DFU has been reported to be 6.3% and 5.5% in the world and Asia, respectively [1]. A study from North India reported that two-thirds of patients with diabetes are “at risk” for DFU, and 9% had prevalent ulcers, of which 20.2% required amputation [2]. There is an increased risk of mortality in patients with DFU as compared to diabetes without ulcers. On long-term follow-up, the five-year and 10-year mortality was 22% and 71%, respectively, in DFU as compared to 3% and 5%, respectively, in diabetes without ulcers [3].

DFU can be classified as neuropathic ulcer, ischemic ulcer, or neuroischemic ulcer. Oxygen is critical for all wound healing processes. It prevents wounds from infection, increases keratinocyte differentiation and re-epithelialization, enhances fibroblast proliferation and collagen synthesis, and promotes wound contraction [4,5]. Blood delivers oxygen to the tissue, and the majority of oxygen delivered is bound to hemoglobin (Hb) in red blood cells (RBCs). Therefore, anemia has the potential to reduce oxygen delivery at the ulcer site and thus hampers the healing process. Anemia is more common in diabetic patients as compared to nondiabetic ones [6,7]. Based on regional studies in India, anemia is highly prevalent in the general population, and its prevalence is reported to be between 43% and 74% in individuals with DM [6,8,9]. The presence of anemia increases the risk of diabetic complications such as peripheral neuropathy and cardiovascular disease [10,11]. In various studies, the prevalence of anemia is reported to be 53%-85% in those with DFU [12-16]. Anemia could be a predictor of amputation, mortality, and DFU severity [16,17].

DFUs or infections in India are different from those in Western countries [18]. In India, the majority of DFUs are chronic, harbor infection, and are neuropathic type as compared to predominantly neuroischemic ulcers in Western countries. Gram-negative bacterial infections are common in India, while in Western nations, gram-positive infection is more common. There are limited studies on anemia in patients with DFU in India. We aim to investigate the prevalence of anemia and its severity in patients with DFU.

## Materials and methods

This is a retrospective study that included hospitalized patients with type 2 diabetes mellitus with DFU of neuropathic or neuroischemic nature from January 2014 to January 2023. Patients with DFU with type 1 DM, pancreatic diabetes, ischemic ulcer without neuropathy, DFU without infection, malignancy, and steroid-induced hyperglycemia, patients who expired during the study period, and patients whose hemoglobin reports were not available were excluded from the study. The indication for hospitalization in DFU was foot infection with systemic manifestation, cellulitis, leukocytosis, worsening hyperglycemia, acidosis, new or worsening azotemia, electrolyte abnormalities, hemodynamic instabilities, failure of outpatient management, unable or unwilling to comply with outpatient-based treatment, peripheral arterial disease, the requirement of surgical procedures (more than minor), need for intravenous therapy (not appropriate as an outpatient), worsening or non-healing ulcer despite standard of care, and severe anemia. This study was approved by the Institutional Ethics Committee of the Institute of Medical Sciences, Banaras Hindu University (number Dean /2022/EC/3402).

Anemia was defined according to World Health Organization (WHO) criteria: hemoglobin (Hb) concentration of <13 g/dL in males and <12 g/dL in females. Anemia was further classified into the following subgroups: mild anemia (male: Hb of 11-12.9 g/dL, female: Hb of 11-11.9 g/dL), moderate anemia (Hb of 8-10.9 g/dL), and severe anemia (Hb of <8 g/dL). Hemoglobin was estimated using a photoelectric colorimeter. It was performed at an in-house laboratory using Mindray Avantor 5-part counter (Shenzhen, Guangdong, China).

The diagnosis of diabetic neuropathy was considered if two or more of the following are present: neuropathic symptoms (decreased or loss of pain sensation, decreased or loss of temperature perception to cold or hot stimuli, numbness, paresthesia, dysesthesia, or burning pain in lower limbs) and signs such as decreased distal sensation, decreased or absent ankle reflex, decreased or absent vibration sensation by 128 Hz tuning fork test or increased vibration perception threshold (>25 V) on biothesiometer and loss of perception to 10 gm Semmes-Weinstein monofilament test. The diagnosis of peripheral arterial disease was considered with symptoms and signs and occlusion in the peripheral artery (>50%) by color Doppler of both lower limbs. Color Doppler was performed if any of the following characteristics were present: history of claudication pain, ankle-brachial index (ABI) of <0.9, any absent peripheral pulses, gangrenous changes, and ulcer not healing within four weeks despite good standard of care. The diagnosis of osteomyelitis was considered when the probe-to-bone test was positive or bone was involved in X-ray imaging. The severity of DFU was classified according to the University of Texas (UT) classification. Infection in DFU was considered by the presence of two or more signs of inflammation (erythema, pain, tenderness, warmth, or induration) and/or having secondary signs of infection such as foul odor, serous exudates, undermined edge, and discolored or friable wound edges. The severity of infection was classified as mild, moderate, and severe as recommended by the International Working Group on the Diabetic Foot (IWGDF) (2019) [19].

Data was collected from the discharge summary report of patients hospitalized with DFU in the endocrinology ward. Patients’ demographic characteristics and history of comorbidities were collected. Biochemical parameters including Hb levels, blood counts, and other blood parameters were collected and entered onto a Microsoft Excel sheet (Microsoft Corp., Redmond, WA, USA) and analyzed. Categorical and continuous variables were represented as percentage proportions and mean±standard deviation (SD), respectively. Linear association between different factors was performed using Pearson’s correlation analysis. Statistical test for significance at 5% level was tested using Chi-square test or Student’s t-test, as appropriate.

## Results

**Figure 1:**
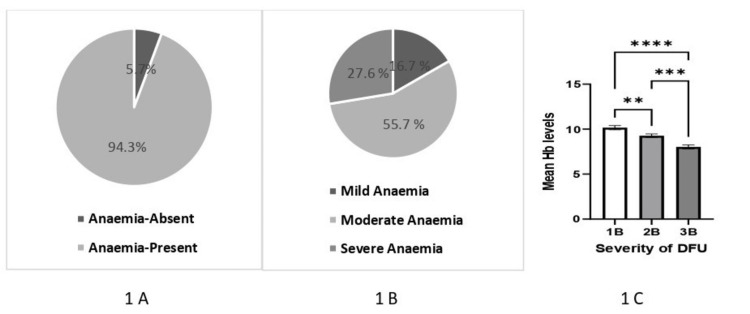
(A) Prevalence of anemia in hospitalized patients with DFU, (B) severity of anemia in DFU, and (C) mean hemoglobin in 1B, 2B, and 3B grade ulcer DFU: diabetic foot ulcer, Hb: hemoglobin

We retrospectively collected and evaluated the data of 323 patients hospitalized with DFU. Twenty-four patients were excluded from the study (type 1 DM (n=2), pancreatic diabetes (n=1), ischemic ulcer without neuropathy (n=2), DFU without infection (n=6), malignancy (n=3), steroid-induced hyperglycemia (n=2), expired (n=4), and patients whose hemoglobin report was not available (n=4)). Table 1 and Table 2 provide the demographic characteristics of the study individuals (n=299) with DFU. The majority (95%) of those with DFU had anemia (Table 2 and Figure 1A). The mean age and gender distribution were similar between those with and without anemia. The duration of DM was significantly higher in the anemia group (8.9±7.2 years versus 5.3±5.1 years; p=0.0394). Neuropathic symptoms were prevalent in 90% of individuals with DFU. A history of trauma was present in 37.5% of cases. The duration of hospitalization was about 1.7 times higher in the anemia group (11.6±5.8 days versus 6.9±3.0 days; p=0.0012). Ankle-brachial index (ABI) was available in 283 (94.68%) patients, and the mean ABI was 1.09±0.20. It was significantly lower in neuroischemic ulcers as compared to neuropathic ulcers (0.99±0.37 versus 1.11±0.14; p=<0.0001).

**Table 1:** Demographic characteristics of the study population SD: standard deviation, BMI: body mass index, DM: diabetes mellitus, DFU: diabetic foot ulcer, HbA1C: hemoglobin A1C, GFR: glomerular filtration rate, TC: total cholesterol, TG: triglyceride, HDL: high-density lipoprotein, LDL: low-density lipoprotein, VLDL: very-low-density lipoprotein

Baseline characteristics		
Number		299
Age (years) (mean±SD)		55.2±10.2
Sex	Male	192 (64.2%)
	Female	107 (35.8%)
BMI (kg/m^2^)		22.1±4.4
Newly diagnosed type 2 DM with DFU		9 (3%)
Duration of DM (years) (mean±SD)		8.7±7.2
Duration of hospitalization (days) (mean±SD)		11.3±5.8
HbA1C (%)		10.9±2.6
Random plasma glucose (mg/dL) at admission		303.1±150.7
Systolic blood pressure (mmHg)		133.8±21.9
Diastolic blood pressure (mmHg)		80±20.4
Pulse rate (beat per minute)		87.7±9.3
Total daily insulin requirement at discharge (unit/day)		34.8±21.6
Biochemical parameters		
GFR (mL/minute/1.73 m^2^)		62.5±28
Total protein (gm/dL)		6.9±1
Albumin (gm/dL)		3±0.6
Sodium (mEq/L)		133.4±6.3
Potassium (mEq/L)		4.6±0.8
TC (mg/dL)		131.9±42.3
TG (mg/dL)		151.5±74.7
HDL (mg/dL)		30.8±14.2
LDL (mg/dL)		77.4±33
VLDL (mg/dL)		30.5±15.1

**Table 2:** Diabetic foot ulcer characteristics and comorbidity at admission DFU: diabetic foot ulcer, UT: University of Texas, IWGDF: International Working Group on the Diabetic Foot, CAD: coronary artery disease, IQR: interquartile range, TIBC: total iron-binding capacity

Baseline characteristics and comorbidities		
DFU classification (UT grade)	1B	83 (27.8%)
	1D	9 (3%)
	2B	108 (36.1%)
	2D	16 (5.3%)
	3B	64 (21.4%)
	3D	19 (6.4%)
Nature of ulcer	Neuropathic ulcer	255 (85.3%)
	Neuroischemic ulcer	44 (14.7%)
Severity of anemia	Mild	47 (16.7%)
	Moderate	157 (55.7%)
	Severe	78 (27.6%)
Severity of infection (IWGDF)	Mild	46 (15.4%)
	Moderate	105 (35.1%)
	Severe	148 (49.5%)
Anemia		282 (94.3 %)
Hypoglycemia		14 (4.7%)
Fever		74 (24.7%)
Neuropathic symptom		270 (90.3%)
History of trauma		112 (37.5%)
Hyponatremia		158 (54.6%)
Gangrene		58 (19.4%)
Amputations		43 (14.4%)
Osteomyelitis		80 (26.8%)
Hypertension		115 (38.5%)
CAD		35 (11.7%)
Diabetic retinopathy		161 (53.8%)
Peripheral artery disease		44 (14.7%)
Urinary tract infection		19 (6.4%)
Primary hypothyroidism		28 (9.3%)
Duration of ulcer (days) (median (IQR))		30 (0-365)
Hemoglobin (gm/dL)		9.3±2.1
Blood transfusion		50 (17.7%)
Iron (mcg/dL)		40.22±23.81
TIBC (mcg/dL)		239.34±67.24
Ferritin (ng/mL)		378.05±141.34

More than half of the population with anemia were of moderate severity, and about one-fourth had severe anemia (Figure 1B). The severity of anemia was associated with the severity of DFU (r=-0.35, confidence interval=-0.45 to -0.25; p value<0.0001**** (Table 3)). There is a significant difference in the mean Hb levels among the patients with varying grades of severity of DFU (1B: Hb=10.17±2.08 gm/dL, 2B: Hb=9.27±2.04 gm/dL, 3B: Hb=8.03±1.829 gm/dL; p value=<0.0001) (Figure 1C and Table 4). Categorical subclassification of anemia into mild, moderate, and severe cases indicated a significant correlation (p<0.0001) with varying grades of DFU. Red blood cell (RBC) counts, mean corpuscular volume (MCV), mean corpuscular hemoglobin concentration (MCHC), lymphocyte counts, albumin, calcium, and high-density lipoprotein (HDL) significantly decreased with the increased severity of DFU (Table 3 and Table 4). The duration of hospitalization, total leucocyte counts, neutrophil counts, and neutrophil-to-lymphocyte ratio (NLR) increased with the severity of DFU (Table 3 and Table 4). There was no significant difference in diabetic duration, HbA1C, duration of ulcer, body mass index (BMI), glomerular filtration rate (GFR), systolic blood pressure (SBP), diastolic blood pressure (DBP), total T4 (thyroxine), thyroid-stimulating hormone (TSH), and total protein in grade 1B, 2B, and 3B (Table 4). Iron study was available in 141 (47.15%) patients and was suggestive of anemia of chronic disorder (mean serum iron=40.22±23.81 mcg/dL, mean total iron-binding capacity (TIBC)=239.34±67.24 mcg/dL, mean ferritin=378.05±141.337 ng/mL) (Table 2). TIBC significantly decreased with increased DFU severity (1B=262.13±61.05, 2B=233.65±71.26, 3B=222.43±74.18; p=0.04). Ferritin significantly increased with increased diabetic foot severity (1B=309.9±70.76, 2B=351.73±94.22, 3B=488.58±170.4; p<0.0001). Serum iron was significantly lower in grade 3B in comparison to grade 1B (3B=32.24±15.65, 1B=46.22±22.21; p=0.003), but there was no difference between grade 1B and 2B and between grade 2B and 3B (Table 4). Twenty-five (8.36%) patients have established chronic kidney disease (CKD), and out of that, only two patients were on maintenance hemodialysis. One hundred thirty-three (44.14%) patients had a GFR of less than 60 mL/minute/1.73 m^2^, although there may be a contribution of acute kidney injury (AKI) to the decreased GFR.

**Table 3:** Correlation of UT grade with blood parameters UT: University of Texas, Hb: hemoglobin, RBC: red blood cell, TLC: total leucocyte count, NLR: neutrophil-to-lymphocyte ratio *p value≤0.05, **p value≤0.01, ***p value≤0.001, ****p value≤0.0001

UT grade versus complete blood counts	Number of XY pairs	R	95% confidence interval	P (two-tailed)
Hb (gm/dL)	299	-0.35	-0.45 to -0.25	<0.0001^****^
RBC (million/mm^3^)	99	-0.30	-0.47 to -0.11	0.0023^**^
TLC (10^3^/mm^3^)	295	0.27	0.16 to 0.38	<0.0001^****^
Neutrophils (%)	295	0.22	0.11 to 0.33	0.0001^***^
Lymphocytes (%)	295	-0.24	-0.34 to -0.12	<0.0001^****^
Monocytes (%)	111	0.03	-0.16 to 0.21	0.795
Basophils (%)	103	0.01	-0.18 to 0.20	0.9003
Eosinophils (%)	106	-0.09	-0.28 to 0.09	0.3268
Platelets (10^3^/mm^3^)	268	-0.07	-0.19 to 0.05	0.2507
NLR	295	0.26	0.15 to 0.36	<0.0001^****^

**Table 4:** Clinical and biochemical parameters according to UT grade UT: University of Texas, BMI: body mass index, GFR: glomerular filtration rate, Hb: hemoglobin, RBC: red blood cell, TLC: total leucocyte count, NLR: neutrophil-to-lymphocyte ratio, MCV: mean corpuscular volume, MCH: mean corpuscular hemoglobin, HbA1C: hemoglobin A1C, TIBC: total iron-binding capacity, ALP: alkaline phosphatase *p value≤0.05, **p value≤0.01, ***p value≤0.001, ****p value≤0.0001

	1B (n=83)	2B (n=108)	3B (n=64)	p value
Age (years)	55.73±11	54.37±9.46	53.44±9.11	0.3667
Duration of hospitalization (days)	9.16±4.71	11.35±5.65	12.95±6.94	0.0003***
Duration of diabetes mellitus (years)	8.95±8.13	7.80±6.06	9.96±7.45	0.1527
Random plasma glucose at admission (mg/dL)	339.9±151.7	314.3±147	292.8±156.1	0.4591
Ulcer duration (days)	48.73±80.44	41.54±44.5	66.59±95.88	0.1364
BMI (kg/m^2^)	22.59±4.77	22.22±4.30	21.55±4.21	0.6065
GFR (mL/minute/1.73 m^2^)	61.65±27.39	65.85±26.5	57.09±32.36	0.2181
Hb (gm/dL)	10.17±2.09	9.27±2.04	8.03±1.83	<0.0001****
RBC (million/mm^3^)	3.62±0.79	3.22±0.75	3.03±0.73	0.0206*
TLC (10^3^/mm^3^)	11.14±5.18	13.05±5.82	14.55±7.50	0.0043**
Neutrophils (%)	70.13±10.21	74.37±11.6	75.64±15.4	0.0095**
Lymphocytes (%)	20.55±8.67	17.14±9.79	15.03±10.95	0.0028**
NLR	4.68±3.70	6.78±5.38	10.15±11.58	0.0001***
MCV (fL)	85.3±5.91	83.97±6.4	81.68±7.80	0.0073**
MCH (pg)	28.24±2.55	28.86±10.7	26.87±3.33	0.0342*
HbA1C (%)	10.56±3.31	11.18±2.48	11.43±2.22	0.4813
Iron	46.22±22.21	41.03±30.98	32.24±15.65	0.059
TIBC	262.13±61.05	233.65±71.26	222.43±74.18	0.04*
Ferritin	309.90±70.76	351.73±94.22	488.58±170.40	<0.0001^****^
ALP (U/L)	236.5±220.6	236.3±143.0	276.5±185.4	0.3562
Total protein (gm/dL)	7.02±0.99	6.82±0.94	6.90±1.0	0.4035
Albumin (gm/dL)	3.3±0.68	3.01±0.59	2.82± 0.61	<0.0001****
Calcium total (mg/dL)	9.13±0.9417	8.81±0.91	8.68±0.85	0.0181*

Hemoglobin was significantly decreased at discharge as compared to at admission (9.3±2.1 gm/dL versus 8.8±1.5 gm/dL; p value=0.01). Total leucocyte counts (TLCs) and neutrophil counts decreased, and lymphocytes increased at discharge in comparison to at admission (TLC=13.18±6.5 103/mm^3^ versus 10.6±7.5 103/mm^3^; p value=0.0003) (neutrophil counts=74.0%±12.3% versus 69.6%±13.2%; p value=0.001) (lymphocyte counts=17.3%±9.8% versus 19.5%±9.3%; p value=0.03). A logistic regression model with retinopathy, peripheral arterial disease, coronary artery disease, and CKD as outcome variables and severe anemia (Hb < 8 g/dL) as a predictor variable was developed, but no relationship was found. It was observed that severe anemic patients were at 68% higher risk (odds ratio (OR)=1.68) of having retinopathy compared to individuals whose Hb level was above 8 g/dL. This relationship was strong but not statistically significant (p=0.054) probably due to the small sample size. Nineteen (6.4%) patients had urinary tract infections, of which two had emphysematous pyelonephritis needing double J stenting.

With respect to the other prevailing symptoms and comorbidities, individuals with anemia had a similar trend in the prevalence of gangrene, amputations, or osteomyelitis as the group of patients without anemia. The prevalence of other comorbidities including hypertension, coronary artery disease (CAD), and diabetic retinopathy was comparable to their prevalence in all DFU patients.

## Discussion

In our study, of the Indian patients with DFU, 94.3% have anemia of varying degrees, suggesting a strong correlation between their coexistence. The prevalence of anemia in DFU was reported from 53.6% to 85.3% in different studies [12-16]. A study from Nigeria found the prevalence of anemia in half of the patients admitted with DFU, and out of that, 48.9% required blood transfusion [15]. In our study, we found a very high prevalence of anemia (94.3%), but blood transfusion was done only in 17.7% of cases. This could be attributed to the financial constraints of the individuals and the limitation of resources to ensure the availability of blood for transfusion. The high prevalence of anemia in our study as compared to the Nigerian study was possibly due to the inclusion of all DFUs associated with infection (100% versus 76.8%), the prolonged duration of ulcer (49.49 versus 39 days), and renal impairment (44.14% versus 19.6%) being more common in our study [15]. Olgun et al. [13] in their retrospective study from Turkey reported the prevalence of anemia to be 85.3%, 56% had iron deficiency anemia, and only 16 patients had anemia of chronic disorder [13]. In our study, a detailed workup of anemia was available in 47.15% of cases and was suggestive of anemia of chronic disorder in the majority of patients (92.6%). None of the patients had isolated absolute iron deficiency anemia. The diagnosis of iron deficiency anemia is challenging in the presence of inflammation, and the possibility of concomitant iron deficiency and vitamin B12 deficiency could not be ruled out in this study [20].

Another common cause of anemia in DFU is CKD. Urea and creatinine levels have a significant negative correlation with Hb levels. This is corroborated by a positive significant correlation with glomerular filtration rate (GFR). At admission, half of the patients had a GFR of <60 mL/minute/1.73 m^2^, although there may be a contribution of acute kidney injury (AKI) to the decreased GFR. In a population-based study, New et al. [21] found that below GFR of 83 mL/minute/1.73 m^2^, for every 1 mL/minute/1.73 m^2^ fall in GFR, there was an associated 0.4 (0.3-0.5) g/L fall in hemoglobin. In another observational study, Margolis et al. [22] found a relationship between the severity of CKD and DFU and concluded that CKD is a risk factor for amputation in DFU. In our study, although we do not see a trend in association with different UT grades, there is a significant correlation with Hb levels, linking GFR to anemia in DFU.

In terms of the type of anemia, the association with grade 1B, 2B, and 3B is suggestive of the prevalence of anemia of chronic disorder. The severity of infection (increased TLC, neutrophils, and NLR, and decreased lymphocytes) was associated with the severity of DFU and the severity of anemia. Further findings in favor of anemia of chronic disorder were decreasing TIBC and increasing ferritin with increasing severity of DFU. During hospitalization of an average of 10.51±5.5days, hemoglobin was significantly decreased despite improvement in infection with treatment. This could be possibly due to blood loss in frequent blood sampling, frequent debridement, low oral intake because of sepsis, and antimicrobial agent during hospitalization. Similarly, Wright et al. [23] reported the decline in hemoglobin and the persistence of anemia in DFU at three months of follow-up. Since the number of individuals with 1D, 2D, and 3D grade DFU is low, we could not substantiate our interpretations of these types of DFU.

In our study, we found that the severity of anemia was associated with the severity of DFU. Similar to our study, a meta-analysis of 15 studies with 2,895 patients demonstrated a clear association between the severity of anemia and the severity of DFU [16]. Previous studies reported that anemia could be a predictor of non-healing ulcers, amputation, and mortality [14-17]. However, in our study, we did not analyze any association because the number of patients without anemia was very low (5.7%). We found a very high prevalence of anemia, and its severity increased with the severity of DFU. The severity of DFU was associated with the duration of hospitalization, RBC counts, TLC, neutrophil counts, lymphocyte count, NLR, MCV, MCHC, albumin, calcium, and HDL. In this retrospective study, we find hyponatremia (serum sodium < 135 mEq/L) in 54.6% of cases at admission. This observation was most probably because of poor glycemic control (HbA1C=10.9%±2.6% and random blood glucose=303.14±150.7 mg/dl), sepsis, renal failure, heart failure, poor oral intake, nausea, and drugs such as diuretics and amitriptyline. The prevalence of gangrene and neuroischemic ulcers was 19.4% and 14.7%, respectively. This difference was possibly due to less sensitivity of color Doppler for small peripheral vessel stenosis or because of septic emboli in neuropathic ulcers.

There are certain limitations in this study. It is a retrospective hospital-based study, and follow-up was not available. The number of patients with neuroischemic ulcers was less. A detailed workup of anemia was not available, and iron studies were done in less than half of the patients.

## Conclusions

The prevalence of anemia in DFU with infection was high. More than three-fourths of patients had moderate to severe anemia. The severity of anemia was associated with the severity of DFU. Most of the ulcers were neuropathic in nature and had moderate to severe grade infection. Hemoglobin was significantly decreased at the time of discharge as compared to on admission despite the optimal management of DFU and infection. The commonest cause of anemia was anemia of chronic disorder secondary to diabetic foot infection. Further prospective studies are needed for a detailed workup of anemia and to explore the impact of the treatment of anemia in DFU healing.
